# Supplementary material for: Enabling new insights from old scans by repurposing clinical MRI archives for multiple sclerosis research
Source: Nat Commun. 2025 Apr 7;16:3149. doi: 10.1038/s41467-025-58274-8 (PMC11976987; doi:10.1038/s41467-025-58274-8)
Supplement: Supplementary file 1 — Supplementary Information [file 41467_2025_58274_MOESM1_ESM.pdf]

Supplemental Methods

Model architecture

The MindGlide model is a 3D-CNN model using hyperparameters obtained from a recent international challenge of the nnU-net model and implemented using the "dynamic Unet architecture (DynUNet)" inside the MONAI framework[11,12,31]. MindGlide takes 3D images with one input channel and outputs 20 segmented channels, each representing a unique label, including the background. The model is structured into several sections: an input block, a series of down-sampling blocks, a series of up-sampling blocks, an output block, multiple deep supervision heads, and skip layers to facilitate information flow across the network. The input block consists of two convolutional layers (Conv3d) with Leaky ReLU activations and Instance Normalization[50]. The down-sampling section consists of four "Unet basic block" units, each composed of two 3D convolution layers with instance normalisation and LeakyReLU activation. The bottleneck again comprises two convolution layers with instance normalisation and LeakyReLU activation, performing the core extraction of features. The up-sampling section consists of five "Unet up block" units. Each upsample block consists of a ConvTranspose3d layer for up-sampling, followed by a basic Unet block, which contains two Conv3d layers with instance normalisation and LeakyReLU activation. The output block consists of a single 3D convolution layer.

The deep supervision heads consist of three convolutional layers, each one performing convolutions to gradually reduce the number of feature channels to the desired number of output channels (20 in our case). This design comprises 43 layers with 30,781,744 trainable parameters. See the Code Availability section for the URL to our code, pre-trained models and the MONAI implementation (https://www.monai.io).[31]

Evaluating MindGlide with "ground truth" manual lesion segmentations cross sectionally and longitudinally

We used two openly available lesion segmentation datasets as ground truth comparators[37,38]. The first dataset, MS-30, had 30 MS patients (23 women) with median age 39 years (range 25-64) and relapse-onset MS[38]. Each patient had pre-processed and co-registered 3D FLAIR, T1-weighted, and T2-weighted images, and a consensus lesion mask generated by three raters (details in [38]). The second dataset, ISBI, was from the 2015 International Symposium on Biomedical Imaging Lesion Challenge[51]. It had a training set of 5 patients, each with four time-separated scans for longitudinal lesion segmentation. Each patient and timepoint had co-registered FLAIR, T1, T2 and proton density data. Two independent raters provided lesion masks, but no consensus mask. We used the most experienced rater (17 years) for cross-sectional analysis. We restricted analysis to FLAIR lesion segmentations and ran MindGlide and SAMSEG on all FLAIR images. For cross-sectional lesion segmentation, we pooled the datasets to create n=50 FLAIR lesion image masks from 35 patients. We assessed lesion load (mm3), voxel-wise spatial metrics (Dice overlap scores, sensitivity, precision) to compare segmentation software against reference masks.

To assess lesion segmentation longitudinally, we used the ISBI dataset to calculate the Intraclass Coefficient (ICC) between rater 1 and rater 2, rater 1 and MindGlide and for completeness, rater 2 and MindGlide. To this end we performed a linear mixed effects model with lesion volume as the dependent variable, rater as a fixed effect and both time and subject as random effects (R software, lme4 package). The ICC was calculated from the variance of the model output and defined as the total rater variance divided by the total rater variance + the residual variance of the model.

Supplemental Results

Lesion Segmentation Analysis

Lesion load was calculated from each software and reference rater mask. Mean lesion loads were 15456.6 ± 14399.9 mm^3 for the ground truth, 14250.3 ± 12447.3 mm^3 for MindGlide and 6296.4 ± 6331.2 mm^3 for SAMSEG. One-way ANOVA revealed a significant Group effect ($F_{(1,48)} = 9.234$, $p < 0.001$) and post hoc pairwise comparisons revealed a significant difference between lesion load calculated from ground truth masks vs SAMSEG (p < 0.001) and MindGlide vs SAMSEG (p < 0.01) (see Figure 3 in the main text) with SAMSEG defining smaller lesions compared to both ground truth masks and MindGlide lesion segmentations.

All spatial metric summary data and statistics are presented in Table 2.

An ICC analysis assessed how well MindGlide performed at segmenting lesions from the same patient across multiple time points. For this, we calculated ICC agreement values for rater 1 vs rater 2, rater 1 vs MindGlide and rater 2 vs MindGlide, with the ICC values being 0.98, 0.97 and 0.96, respectively.

Treatment Effect Analysis on Percentage Brain Volume Change

We calculated treatment effects on percentage brain volume change (PBVC) for every MRI contrast in our PPMS dataset (Supplemental Figure 3). Annualised PBVC in T1-weighted images was -0.91% in the placebo group and -0.85% in the treatment group (p-value = 0.572). In T2-weighted images PBVC was -0.65% in the placebo group and -0.56% in the treatment group. (p-value = 0.279). In FLAIR images PBVC was -1.22% in the placebo group and -1.12% in the treatment group (p-value = 0.442) and in PD images PBVC was -0.95% in the placebo group and -0.81% in the treatment group, respectively (p-value = 0.232). Despite showing slower brain volume loss over time in the treatment groups across all MRI contrasts, all the results were statistically not significant.

Supplemental Tables

Supplemental Table 1. Data sources used for training and external testing of MindGlide.

Trial	Included in this study*
**Training datasets**	
ADVANCE [16]	959
ASCEND [17]	997
BRAVO [18]	494
DCE (DEFINE, CONFIRM, ENDORSE) [19–21]	53
EXPAND [3]	1004
INFORMS [23]	344
SPI2 [24]	373
Observational UCL cohort (PITMS cohort, adults with MS)	23
**External validation**	
Paediatric MS	161

ORATORIO[2]	699
MS-STAT [25]	141

* We sampled the training data from the International Progressive MS Alliance data repository at the Montreal Neurological Institute.

Supplemental Table 2. Cross-software comparison of lesion segmentation (MindGlide and SAMSEG).

	**Ground Truth**	**MindGlide**	**SAMSEG**	**WMH-Synthseg**
Median Lesion Load (mm^3)	11823 (3200 - 22943)	10614 (4353 - 23155)	4119 (1336 – 10281)	8648 (3938 – 11974)
Dice		0.61 (0.45 – 0.72)	0.50 (0.19 – 0.62)	0.38 (0.25 – 0.44)
Sensitivity		0.60 (0.43 – 0.76)	0.35 (0 .11 – 0.49)	0.30 (0.23 – 0.40)
Precision		0.63 (0.44 – 0.80)	0.86 (0.60 – 0.92)	0.47 (0.22 – 0.63)

Caption: Parentheses show interquartile range [IQR]. Total n=50.

Supplemental Table 3. Correspondence between MindGlide labels and Neuromorphometrics' atlas.

MindGlide label	Neuromorphometrics' atlas label
Brain stem	Brain_Stem
Cerebellum	Cerebellum_Exterior, Cerebellum_White_Matter, Cerebellar_Vermal_Lobules_I-V, Cerebellar_Vermal_Lobules_VI-VII, Cerebellar_Vermal_Lobules_VIII-X
Corpus callosum	Corpus_Callosum
CSF	Non-ventricular_CSF
DGM	Accumbens_Area, Amygdala, Pallidum, Putamen, Caudate, Thalamus_Proper, Basal_Forebrain
Frontal lobe GM	Claustrum, FO_frontal_operculum, FRP_frontal_pole, GRe_gyrus_rectus, LOrG_lateral_orbital_gyrus, MFC_medial_frontal_cortex, MFG_middle_frontal_gyrus, MOG_middle_occipital_gyrus, MOrG_medial_orbital_gyrus, MSFG_superior_frontal_gyrus_medial_segment, OpIFG_opercular_part_of_the_inferior_frontal_gyrus, OrIFG_orbital_part_of_the_inferior_frontal_gyrus, PIns_posterior_insula, POrG_posterior_orbital_gyrus, PrG_precentral_gyrus, SCA_subcallosal_area, SFG_superior_frontal_gyrus, MC_supplementary_motor_cortex, TrIFG_triangular_part_of_the_inferior_frontal_gyrus
Lateral Ventricle	Lateral_Ventricle, Ventricular_Lining, Inf_Lat_Vent
Limbic cortex GM	ACgG_anterior_cingulate_gyrus, AIns_anterior_insula, AOrG_anterior_orbital_gyrus, Ent_entorhinal_area, MCgG_middle_cingulate_gyrus, PCgG_posterior_cingulate_gyrus, PHG_parahippocampal_gyrus
Occipital lobe GM	Calc_calcarine_cortex, Cun_cuneus, IOG_inferior_occipital_gyrus, LiG_lingual_gyrus, OCP_occipital_pole, OFuG_occipital_fusiform_gyrus, SOG_superior_occipital_gyrus, AnG_angular_gyrus, MPoG_postcentral_gyrus_medial_segment, MPrG_precentral_gyrus_medial_segment, PCu_precuneus, PoG_postcentral_gyrus, SMG_supramarginal_gyrus
Optic Chiasm	Optic_Chiasm
Parietal lobe GM	PO_parietal_operculum, SPL_superior_parietal_lobule
Pons	Pons

Temporal lobe	FuG_fusiform_gyrus, Hippocampus, ITG_inferior_temporal_gyrus, MTG_middle_temporal_gyrus, PP_planum_polare, PT_planum_temporale, STG_superior_temporal_gyrus, TMP_temporal_pole, TTG_transverse_temporal_gyrus
Third and fourth ventricle	3rd_Ventricle, 3rd_Ventricle_(Posterior_part), 4th_Ventricle
Ventral dc	Ventral_DC
White_matter	Parietal_White_Matter, Temporal_White_Matter, Insula_White_Matter, Cingulate_White_Matter, Frontal_White_Matter, Occipital_White_Matter

Lesions are not included because they are not part of the Neuromorphometrics' atlas.

Abbreviations: GM, grey matter; DGM, deep grey matter; CSF, cerebrospinal fluid; DC, Diencephalon;

Supplemental Table 4: Median running time of a MindGlide, SAMSEG and WMH-Synthseg on a single MRI contrast during inference.

**Model**	**Runtime (IQR)**	**GPU / CPU**	**GPU Memory**	**Maximum GPU Memory used during Inference**
Mindglide	37 seconds (34 - 40)	GPU: NVIDIA Quadro RTX 6000	24 GB	7850 MiB
SAMSEG	42 minutes (28 – 57)	CPU: Intel(R) Xeon(R) Gold 6140 CPU @ 2.30GHz	-	-
WMH-Synthseg	15 minutes (10 – 19)	CPU: Intel(R) Xeon(R) Gold 6140 CPU @ 2.30GHz	-	-

Runtime was analyzed across 10109 scans and maximum used GPU memory across 90 scans. MindGlide runs on GPU while we performed our analysis with WMH-Synthseg and SAMSEG on the default setting, which uses CPU. Additionally, we did not calculate the time for longitudinal SAMSEG because it analyses MRI scans of multiple timepoints of a patient at once which prolongs the computing time.

Supplemental Table 5: Power analysis based on treatment effects derived from MindGlide's segmentation volumes using a single contrast.

Dataset for treatment effect estimation (PPMS = 699 subjects SPMS = 141 subjects)	Resolution	Contrast	Region	Participants required for 80% power
PPMS	2D	T2	Lesion	76
PPMS	2D	FLAIR	Lesion	62
PPMS	2D	PD	Lesion	94
PPMS	2D	T1	CGM	2166
PPMS	2D	T2	CGM	420
PPMS	2D	FLAIR	CGM	1244
PPMS	2D	PD	CGM	1375
PPMS	2D	T1	DGM	1882
PPMS	2D	T2	DGM	3180
PPMS	2D	FLAIR	DGM	1506
PPMS	2D	PD	DGM	562
SPMS	2D	T2	Lesion	228
SPMS	2D	T1	CGM	496
SPMS	2D	T2	CGM	120
SPMS	3D	T1	CGM	128
SPMS	2D	T1	DGM	592
SPMS	2D	T2	DGM	126
SPMS	3D	T1	DGM	88

We derived treatment effects from the SPMS trial for SPMS and from the PPMS trial for PPMS on the same region and the contrast noted in each row.

Abbreviations: PPMS, Primary Progressive MS; SPMS, Secondary Progressive MS; CGM, cortical grey matter; DGM, deep grey matter;

Supplemental Figures

Supplemental Figure 1. Correlation of segmentations between 3D and 2D acquisitions.

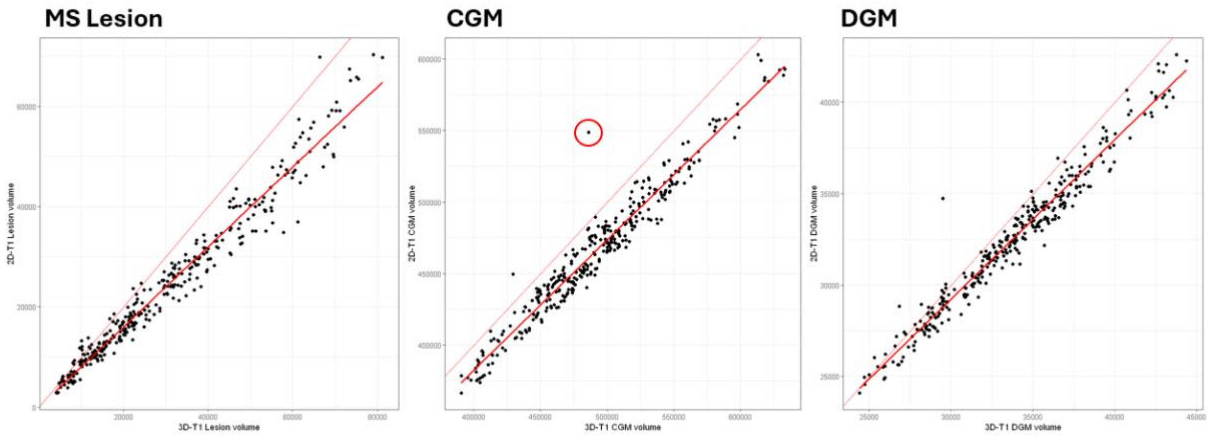

Caption: Correlation of volumes derived from 3D-T1 (1x1x1mm) and 2D-T1 (1x1x3mm) MRI sequences for lesions, cortical grey matter (CGM), and deep grey matter (DGM) visualised using scatter plots. MindGlide demonstrates consistent volume estimation across varying image resolutions. The thicker red line represents the regression line, and the thinner red line represents the line of identity. The outlier (red circle) in the CGM plot was caused by a cropped brain in the 3D-T1 scan, resulting in incomplete segmentation. Data source: SPMS dataset (N=141)

Pairwise comparison of PBVCs of each contrast

Supplemental Figure 2. Scatter Plots of PBVC Measurements across different MRI contrasts

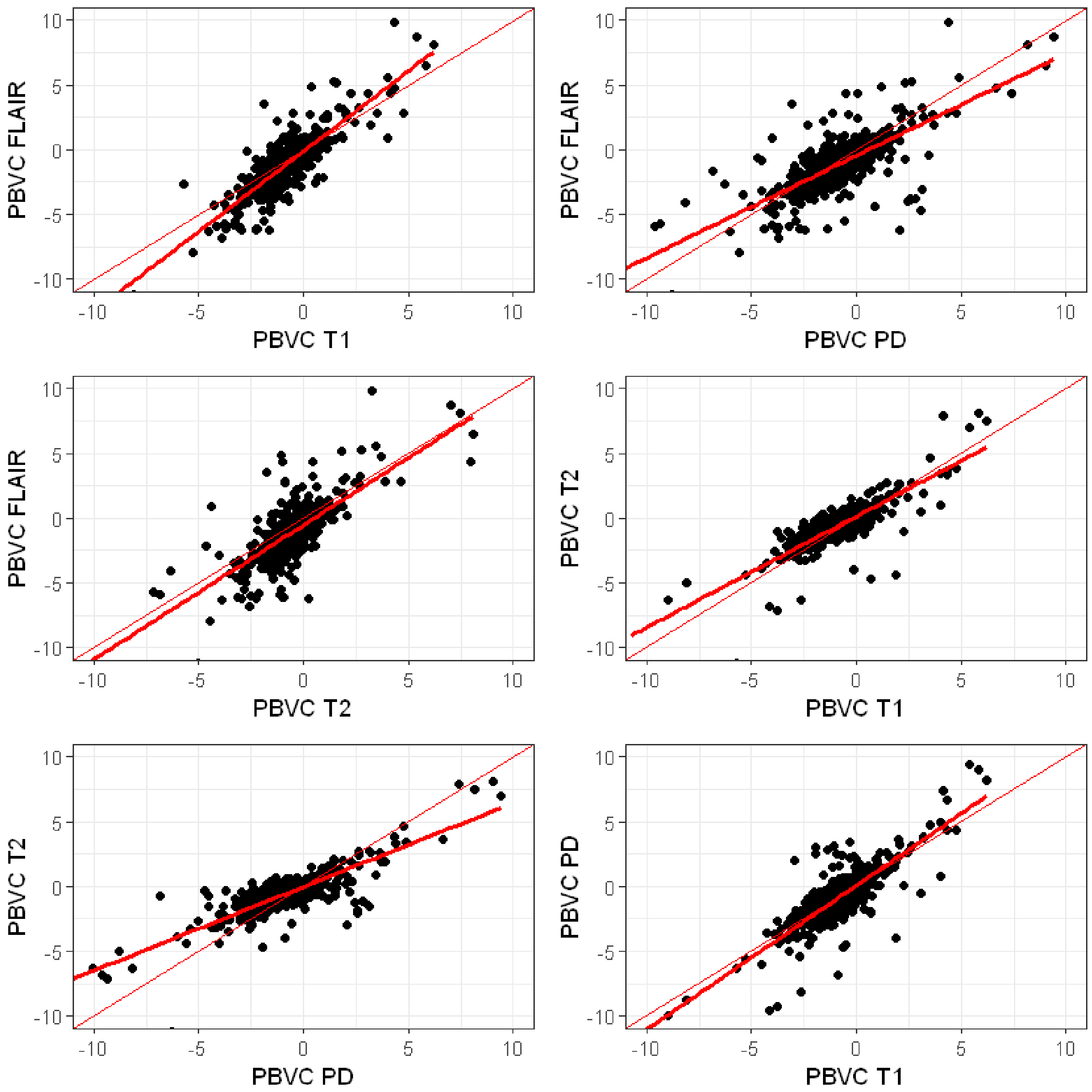

Scatter plots visualising pairwise comparisons of Percent Brain Volume Change (PBVC) per year measured from different MRI contrasts in the PPMS dataset: T1-weighted, T2-weighted, Proton Density (PD), and FLAIR. Each plot displays the relationship between two distinct MRI contrast measurements, highlighted with points representing individual observations. The thinner line in each plot represents a line of unity (slope = 1, intercept = 0), serving as a

reference for perfect agreement between the measurements from the two MRI contrastss. Linear regression lines (the thicker red line) indicated the regression line fitted to data. PPMS dataset. N = 680

Supplemental Figure 3: Brain atrophy in treated and control arms.

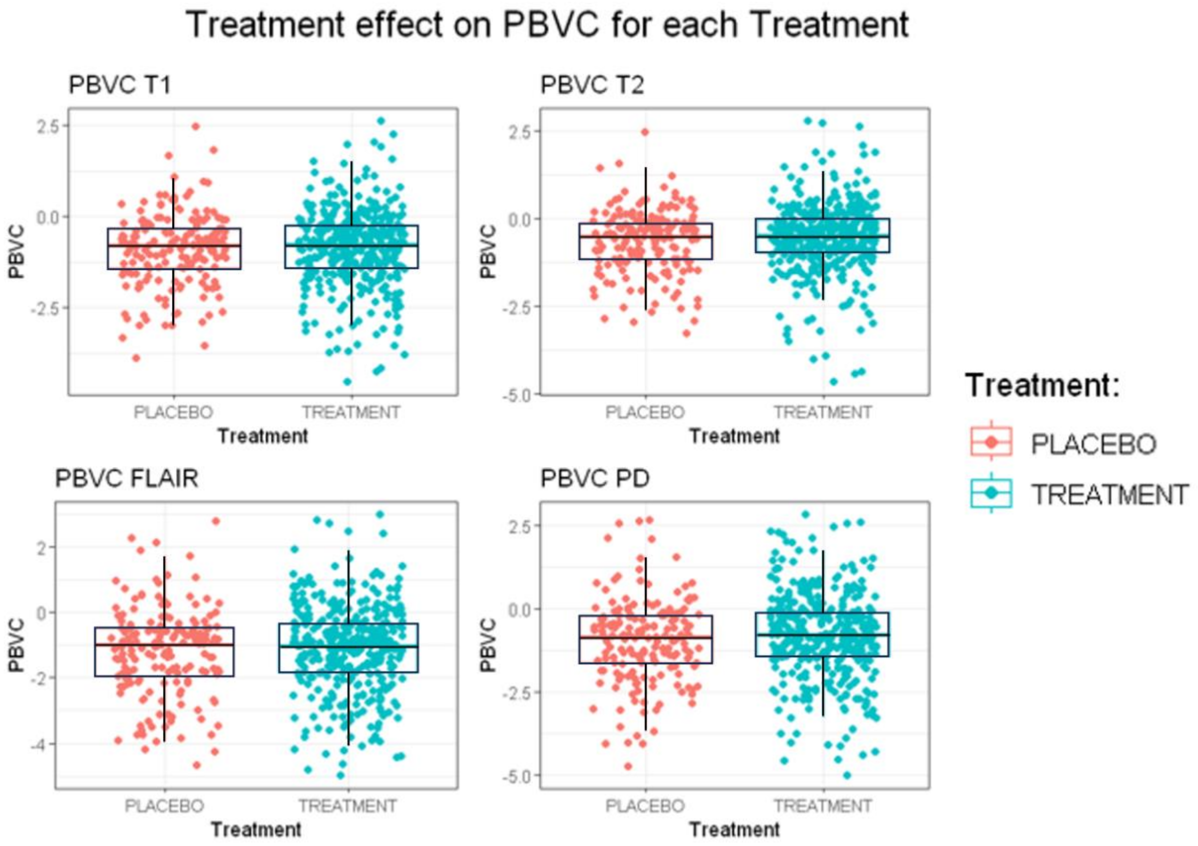

Supplemental Figure 3 shows boxplots and individual data points, illustrating the distribution and variability of PBVC per year measurements across four different MRI contrasts in the PPMS dataset: T1-weighted, T2-weighted, FLAIR, and Proton Density (PD). The data is categorized by two treatment groups, to evaluate the treatment effect on PBVC. Red points indicate the placebo group, while turquoise points represent the treatment group. We found less atrophy in the treatment group compared to the placebo group in every contrast. However, none of these were statistically significant. To enhance the graph's visual clarity, 14 data points classified as outliers have been intentionally omitted from the visualization. Only patients with a follow-up of at least 2 years were used. N = 576.

Supplemental Figure 4 Real-World Dataset: Distribution of Slice Thickness by Contrast

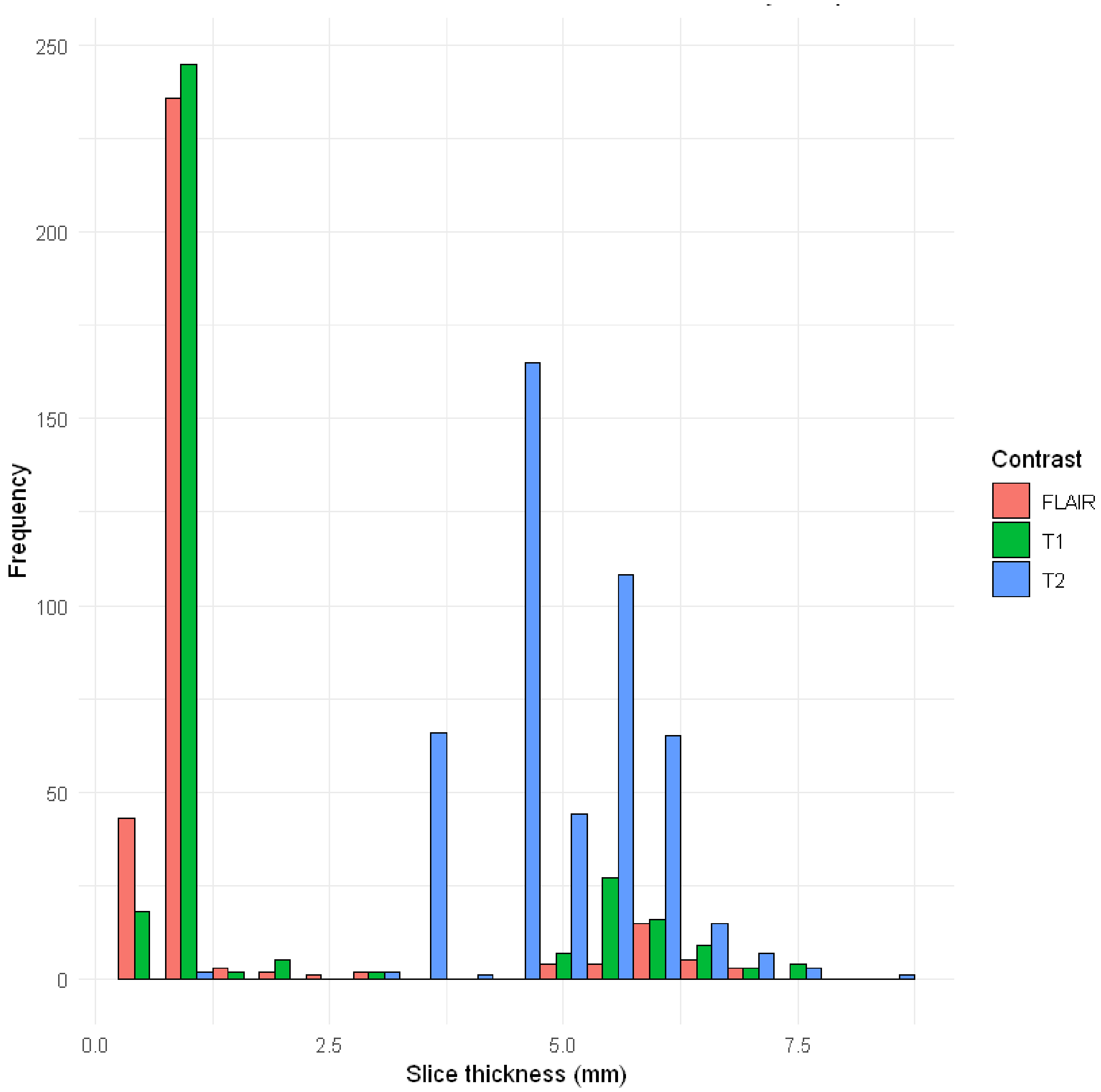

Caption: This histogram shows the frequency of slice thickness (in mm) across the three MRI contrasts in our real-world dataset: FLAIR (red), T1-weighted (green), and T2-weighted (blue). FLAIR and T1 contrasts both have a median slice thickness of 1.0 mm, with minimum values of 0.43 mm and 0.45 mm, respectively, and maximum values of 7.20 mm and 7.65 mm, respectively. T2-weighted images exhibit a broader distribution, with a median slice thickness of 4.8 mm, a minimum of 0.98 mm, and a maximum of 8.50 mm. The distribution highlights a predominance of thinner slices (0-1 mm) in FLAIR and T1-weighted images, while T2-weighted images show peaks around thicker slices.
